# Drought-Tolerant Plant Growth-Promoting Rhizobacteria Associated with Foxtail Millet in a Semi-arid Agroecosystem and Their Potential in Alleviating Drought Stress

**DOI:** 10.3389/fmicb.2017.02580

**Published:** 2018-01-11

**Authors:** Xuguang Niu, Lichao Song, Yinong Xiao, Weide Ge

**Affiliations:** ^1^College of Land and Environment, Shenyang Agricultural University, Shenyang, China; ^2^National Engineering Laboratory for Efficient Utilization of Soil and Fertilizer Resources, Shenyang, China; ^3^Institute of Crops, Liaoning Academy of Agricultural Sciences, Shenyang, China

**Keywords:** 1-aminocyclopropane-1-carboxylic acid (ACC) deaminase, plant growth-promoting rhizobacteria (PGPR), foxtail millet (*Setaria italica* L.), drought stress, semi-arid region

## Abstract

The application of plant growth promoting rhizobacteria (PGPR) to agro-ecosystems is considered to have the potential for improving plant growth in extreme environments featured by water shortage. Herein, we isolated bacterial strains from foxtail millet (*Setaria italica* L.), a drought-tolerant crop cultivated in semiarid regions in the northeast of China. Four isolates were initially selected for their ability to produce ACC deaminase as well as drought tolerance. The isolates were identified as *Pseudomonas fluorescens, Enterobacter hormaechei*, and *Pseudomonas migulae* on the basis of 16S rRNA sequence analysis. All of these drought-tolerant isolates were able to produce EPS (exopolysaccharide). Inoculation with these strains stimulated seed germination and seedling growth under drought stress. *Pseudomonas fluorescens* DR7 showed the highest level of ACC deaminase and EPS-producing activity. DR7 could efficiently colonize the root adhering soil, increased soil moisture, and enhance the root adhering soil/root tissue ratio. These results suggest drought tolerant PGPR from foxtail millet could enhance plant growth under drought stress conditions and serve as effective bioinoculants to sustain agricultural production in arid regions.

## Introduction

Drought stress is one of the major agricultural problems reducing crop yield in arid and semiarid regions of the world. Changes in mean global air temperature and precipitation patterns are leading to longer drought periods and more extremely dry years, and more severe drought conditions will hinder food production in some countries (Lau and Lennon, 2012). At present, strategies to increase the ability of plants to tolerate drought stress involve the use of water-saving irrigation, traditional breeding, and genetic engineering of drought-tolerant transgenic plants. Unfortunately, these methods are highly technical and labor-intensive, and thus difficult to apply in practice.

One alternative for growing plants under dry conditions is the use of plant growth promoting rhizobacteria (PGPR). PGPR are a group of bacteria that can be found in the rhizosphere in association with plant root systems, both at the root surface and in endophytic associations, and which can either directly or indirectly facilitate plant growth in optimal, biotic, or abiotic stress conditions (Bashan and Holguin, 1998; Cassán et al., 2009). Known mechanisms used by PGPR include nitrogen fixation for plant use, phytohormone production (including auxins, cytokinins, and gibberellins), solubilization of mineral phosphates, and iron sequestration by bacterial siderophores (Glick et al., 1999). In addition, PGPR are linked to catabolism of molecules related to stress signaling, such as bacterial 1-aminocyclopropane-1-carboxylate (ACC) deaminase. Many PGPR have been shown to alleviate drought stress effects in plants by reducing plant ethylene levels that are usually increased by unfavorable conditions (Mayak et al., 2004; Arshad et al., 2008). However, the ability of inoculated bacteria to survive, outcompete with the native microflora, and colonize in the rhizosphere remains to be a critical step for successful application (Bashan, 1998) especially in drought-stressed soils since microorganisms not adapted to high water tension will die under these adverse conditions (Van Meeteren et al., 2008). The drought-tolerant rhizobacteria could thus be advantageous over others to thrive in a new drought environment in the sufficient numbers to deliver beneficial effects on plants.

The rhizobacteria assemblages of many agricultural crops have been studied, and the use of PGPR holds promise for plant growth promotion and alleviation of plant drought stress (Mayak et al., 2004; Zahir et al., 2008; Sandhya et al., 2009). However, the drought-tolerant bacteria associated with crop species which are naturally adapted to drought, such as foxtail millets, have not been explored.

Foxtail millet (*Setaria italica* L.) is a particularly important food and fodder grain crop grown in arid, uncultivable, and marginal lands (Lata et al., 2013). It has a high carbohydrate, protein, starch, fat, and fiber content, and is tolerant to drought and salt stress. It is thus a suitable crop for areas that are constantly subjected to drought, such as west Liaoning in China, which has a semi-arid climate and annual rainfall ranging between 424 and 613 mm (Liu et al., 2013). The adaptation of millet to water deprivation has been ascribed to its relatively small leaf area, the cell arrangement in its epidermis, its thick cell walls, and its ability to form a dense root system (Li, 1997). These characteristics have evolved over a long period of time through natural selection. Drought-resistant plants also benefit from association with interacting species, especially those in diverse soil microbial communities, that respond rapidly to environmental changes (Lau and Lennon, 2012). Rhizobacteria associated with foxtail millet in this area constantly face water deprivation, and have presumably adapted to drought stress conditions, and likely contribute to the adaptation of their associated host plants to drought stress. Recent research with 16S/18S/ITS amplicons and metagenomic sequencing demonstrated that foxtail millet enriched specific bacteria and functions in the rhizoplane (Jin et al., 2017). Foxtail millet might be a useful source of effective drought-tolerant bacterial inoculant with plant growth-promoting potential in arid soils.

Herein, we report the isolation of drought-tolerant plant growth-promoting rhizobacteria associated with foxtail millet in semiarid land in west Liaoning Province, Northeast China, and evaluation of their PGP activities under drought stress. The results suggest PGPR have great potential for biotechnological applications in drought-stressed agricultural systems.

## Materials and Methods

### Sampling and Isolation of Root-Associated Bacteria

Healthy plant samples of foxtail millet (*Setaria italica* L.) collected in July from a semiarid region of the Jianping locality (41°40’28”N, 119°63’34”E) in west Liaoning province (NE China). The area is characterized by a semiarid Mediterranean climate with annual average rainfall ranging from 424 to 613 mm (Liu et al., 2013). The soil was classified as a typical cinnamon-colored soil. The main characteristics of the soil were the following: pH = 8.8, total organic carbon = 12.15 g kg^-1^, available nitrogen = 40.11 mg kg^-1^, available phosphorus = 356.07 mg kg^-1^, and available potassium = 221.40 mg kg^-1^. The humidity content (H%) of soil at the time of sampling was 6.2%. Each plant sample was immediately placed in a sterile plastic bag, transported to the laboratory in an ice cooler, and stored at 4°C.

For root-associated bacteria isolation, bulk soil was removed by gently shaking the plants, and root samples were aseptically crumbled into smaller fragments and macerated using a sterile mortar and pestle with sterile distilled water. Tissue extracts were serially diluted, and the appropriate dilutions were spread onto different isolation media as follows: nutrient agar, King’s B medium (King et al., 1954), R_2_A medium (Reasoner’s 2A agar) (van der Linde et al., 1999). Plates were incubated at 28 ± 2°C, and a representative colony was picked and transferred to fresh nutrient agar medium for further studies.

### Screening for ACC Deaminase Activity

1-Aminocyclopropane-1-carboxylate deaminase activity of bacterial isolates was screened based on the ability to use ACC as a sole nitrogen source. Isolates were spot inoculated on DF salts minimal agar medium (Dworkin and Foster, 1958) supplemented with 3 mM ACC instead of (NH_4_)_2_SO_4_ as nitrogen source. ACC deaminase activity of cell-free extracts under non-stress or drought stress (-0.30 MPa) conditions was determined by measuring the production of α-ketobutyrate (α-KB) that is generated by the cleavage of ACC by ACC deaminase (Penrose and Glick, 2003). After determining the amount of protein and α-KB, the enzyme activity was expressed as micromoles of α-KB per mg of protein per hour.

### Screening for Drought Tolerance

Trypticase soya broth of differing water potential (0, -0.05, -0.30, and -0.73 MPa) was prepared by adding the appropriate concentrations of PEG 6,000 (Michel and Kaufmann, 1973), inoculated with 1% of exponentially grown bacterial cultures, and then incubated on a shaker (120 rpm) at 28°C. The cell growth was estimated every 3 h by measuring the optical density at 600 nm using a spectrophotometer (SP-721; Shanghai Spectrum Instruments Co., Ltd., China).

### Amplification of 16S rRNA and *acdS* Gene

The 16S rRNA gene was amplified by PCR using the universal primers 27F 5′-AGA GTT TGA TCC TGG CTC AG-3′ and 1492R 5′-GGT TAC CTT GTT ACG ACT T-3′ under standard conditions. The *acdS* gene was amplified by PCR using degenerate primers ACCf2 (5′-GCA ACA AGA CGC GCA AGY TNG ART AYN T-3′) and ACCr (5′-GTG CAT CGA CTT GCC CTC RWA NAC NGG RT-3′) (Li, 2011). Primers annealed at positions 146 and 900 of the *acdS* reference nucleotide sequence of *Pseudomonas putida* WU4, corresponding to an expected amplification product of approximately 754 bp. PCR reactions were carried out in a 25 μL volume of reaction mixture containing 1× reaction buffer, 2.5 mM dNTP mixture, 10 pM of each primer, Taq DNA polymerase (1 U) (Tiangen Biotechnology Ltd., Beijing, China), and 25 ng of template DNA. The 16S rRNA and *acdS* gene sequences were determined by PCR direct sequencing (Sangon Biotechnology Ltd., Shanghai, China). The obtained sequences of 16S rRNA and *acdS* were compared with published sequences and submitted to GenBank. Phylogenetic analysis of *acdS* gene sequences was performed with MEGA version 4.0 (Tamura et al., 2007). Phylogenetic tree was constructed using the neighbor-joining method, and bootstrap analyses were performed (*n* = 1,000).

### Determination of Other PGP Traits of the Isolates

Four drought-tolerant ACC deaminase-producing isolates were tested *in vitro* for other PGP properties. The N-fixation ability was determined by observing growth on N-free semi-solid JNFb medium (Baldani et al., 1992; Döbereiner et al., 1995), and the *nif*H gene was also amplified using primers PolF (5′-TGC GAY CCS AAR GCB GAC TC-3′) and PolR (5′-ATS GCC ATC ATY TCR CCG GA-3′) (Qin et al., 2014). To determine phosphate solubilization, 5 μL of an overnight bacterial culture was spotted on Pikovskaya’s agar plates (Pikovskaya, 1948) containing 2% tri-calcium phosphate and incubated at 28°C for 24–72 h, and the appearance of a solubilization zone around bacterial colonies was observed. IAA production was examined using the colorimetric method described by Sheng et al. (2008). Polysaccharide production was observed using the spot plate method on RCV-sucrose medium (yeast extract, 0.1 g liter^-1^; super salts solution, 50 ml liter^-1^; phosphate buffer, 15 ml liter^-1^) (Amellal et al., 1998) containing 40 g L^-1^ sucrose. For the detailed quantitative determination of EPS production, the protocol described by Ali et al. (2014) was carried out. The EPS production was expressed as mg of total carbohydrate per mg of protein and the experiment was conducted five times. For siderophore production, 1 μL of bacterial culture grown overnight in Luria broth was spotted on Chrome Azurol S agar plates (Ames-Gottfred et al., 1989). The appearance of an orange halo around bacterial colonies was observed after incubation for 48 h at 28°C (Ali et al., 2014).

### Effects of Selected Strains on Seed Germination under Drought Stress

Foxtail millet (*Setaria italica* L. cv. Liaogu 2) seeds were rinsed in tap water, then surface-sterilized with 1% sodium hypochlorite for 20 min. For bacterial inoculation, overnight cultures were centrifuged at 11,000 × *g* for 20 min, the pellet was resuspended in phosphate buffered saline (PBS), and the optical density was adjusted to 0.6 (˜10^8^ colony forming units, cfu). Surface-sterilized seeds were soaked at room temperature for 10 h in bacterial suspensions (1 mL) and planted in 0.8% (wt/vol) water agar with different water potentials (0, -0.30, -0.49, -1.03 MPa) prepared by adding the appropriate concentrations of PEG 6,000. Control seeds were treated with sterile distilled water only. Five replications of 50 seeds were planted. Seeds were incubated at 27 ± 1°C, and germination was measured after 3 days.

### Effects of Bacterial Inoculation on Plant Growth Promotion under Drought Stress

Foxtail millet seeds (*Setaria italica* L. cv. Liaogu 2) were surface-sterilized and pre-germinated on sterile filter paper in petri plates. After 3 days, uniform sized seedlings were selected and planted in autoclaved culture boxes (7 cm × 8 cm) containing 200 g of air-dried, sieved soil as described above. After 1 week, seedlings were fertilized once with 1/5 Murashige and Skoog (MS) medium (Murashige and Skoog, 1962). Three days after fertilization, some of the seedlings were treated with 40 mL of bacterial suspension (OD_600_ = 0.6), while others were treated with distilled water. Both inoculated and uninoculated treatments were replicated 20 times, and each treatment had three plants per pot. Seedlings were maintained in a growth chamber under a 16 h:8 h light:dark cycle with respective temperatures of 25 and 18°C. Three weeks after the seedlings were inoculated, water stress was induced in five replicates by discontinuing watering. After non-inoculated plants began to show symptoms (wilting), seedlings were harvested (after 9 days of water stress).

### Harvesting, Enumeration of Inoculated Bacteria, and Determination of the RAS/RT Ratio

Fifteen plantlets per treatment were removed from the growth box, colonization of rhizosphere soil by inoculated strains was determined 10 days, 21 days, or 30 days after inoculation using serial dilution plating technique. The entire soil–root system was taken from the pot and agitated gently to remove the bulk soil. Roots were washed by being dipped in sterile water to separate root adhering soil (RAS) from root tissue (RT). A 1 mL aliquot of the supernatant was serially diluted in distilled water and plated on DF minimal medium containing ACC as the sole N source. Plates were incubated at 28°C for 4–5 days, and colonies were counted. To verify that the counted colonies represented the inoculated strain, 12 colonies were randomly selected and checked for their genetic fingerprint by Enterobacterial repetitive intergenic consensus (ERIC)-PCR. The abundance of culturable ACC deaminase-producing bacteria was expressed in terms of the log of colony forming units (cfu) per gram of rhizosphere soil. The root, shoot, and rhizosphere soil dry mass were recorded after drying the remaining samples at 105°C, the soil moisture (SM) was calculated as SM = (W1 - W2)/W1 × 100%, where W1 and W2 were the fresh weight and dried weight of soil and RAS/RT ratio were calculated according to Sandhya et al. (2009).

### Statistical Analysis

Statistical analysis was conducted by using analysis of variance statistical package for social sciences software 19.0, and means were compared using Duncan’s multiple range test; *P* ≤ 0.05 was considered significant. The results were expressed as mean ± SD.

## Results

### Isolation and Screening for ACC Deaminase

A total of 110 bacterial strains were isolated from the roots and rhizosphere soil of foxtail millet, of which 14 strains grew on DF minimal salts medium with ACC serving as the sole nitrogen source, indicating ACC deaminase activity. The ACC deaminase enzyme activity of these isolates was assayed by quantifying the amount of α-KB produced during the deamination of ACC. These isolates showed different levels of ACC deaminase activity based on the results of the quantitative assays (**Table 1**). The highest ACC deaminase activity was exhibited by isolate DR11 (39.40 ± 0.68 μmol α-KB/mg Pr⋅h), followed by DR7 (24.56 ± 2.24 μmol α-KB/mg Pr⋅h), DR30 (9.66 ± 1.57 μmol α-KB/mg Pr⋅h), and DR16 (9.19 ± 0.81 μmol α-KB/mg Pr⋅h).

**Table 1:** ACC deaminase activity of isolated bacteria associated with Millet.

No. of	Genera	Nearest type strain	Similarity	ACC deaminase
isolates			(%) of the	activity in μmol
			16S rRNA gene	α-KB/(mg Pr⋅h)
DR1	*Pseudomonas*	*P. fluorescens* (CP015225)	100	8.22 ± 1.97c
DR7		*P. fluorescens* (FJ407181)	100	24.56 ± 2.24d
DR11		*P. fluorescens* (HQ888871)	99	39.40 ± 0.68e
DR35		*P. migulae* (EU111689)	100	4.79 ± 0.38b
DR45		*P. migulae* (KU258279)	99	3.24 ± 0.58ab
DR68		*P. extremorientalis* (KC329818)	100	4.81 ± 0.73b
DR16	*Enterobacter*	*E. hormaechei* (KU867635)	100	9.19 ± 0.81c
DR30		*E. asburiae* (GQ496664)	99	9.66 ± 1.57c
DR10	*Pantoea*	*P. ananatis* (KF254661)	100	1.89 ± 0.09a
DR89		*P. Agglomerans* (*KU355542*)	100	3.50 ± 0.37ab
DR23	*Klebsiella*	*K. oxytoca* (KT719222)	100	8.17 ± 1.74c
DR59	*Arthrobacter*	*A. pascens* (KJ191024)	99	7.93 ± 0.33c
DR95		*A. oryzae* (KT369925)	99	2.07 ± 0.33a
DR93	*Ochrobactrum*	*O. intermedium* (KX832724)	99	3.29 ± 0.43ab

*Values are presented as the mean of replicates ± SD. Values followed by different lowercase letters are significantly different according to the Duncan’s range test (*P* = 0.05) in all treatments.*

After DNA extraction and PCR amplification, the 14 ACC deaminase-producing strains were identified by sequencing of the 16S rDNA gene. The 16S rDNA gene sequence similarity (99–100%) shared with the closest type strains is presented in **Table 1**. Based on the sequence of the 16S rRNA gene, these strains represent six different genera: *Pseudomonas* (six isolates), *Enterobacter* (two isolates), *Pantoea* (two isolates), *Arthrobacter* (two isolates), *Klebsiella* (one isolate), and *Ochrobactrum* (one isolate).

### Growth of Isolated Strains under Drought Stress

**FIGURE 1:**
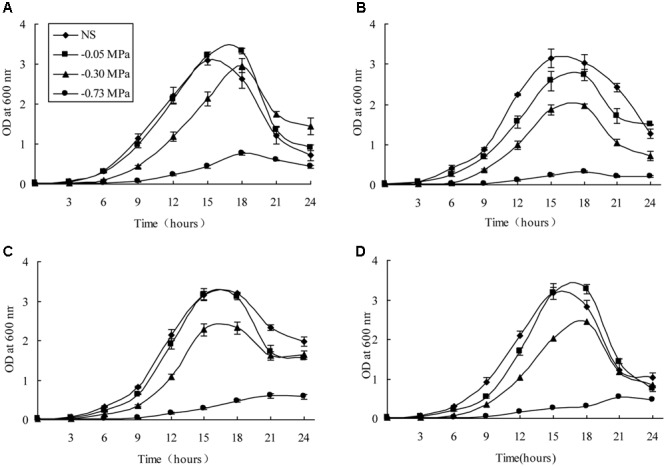
Growth patterns of the four rhizobacteria under non-stressed (NS) and drought-stressed conditions of differing matric potential. **(A)** DR7, **(B)** DR11, **(C)** DR16, **(D)** DR35. Error bars show standard deviations of mean values.

The 14 ACC deaminase-producing bacteria were screened for drought tolerance using polyethylene glycol 6000 (PEG 6,000). The growth of all 14 isolates was affected due to matric stress caused by PEG 6,000, of which four isolates (*Pseudomonas fluorescens* DR7, *Pseudomonas fluorescens* DR11, *Enterobacter hormaechei* DR16, and *Pseudomonas migulae* DR35) were able to grow at a minimum water potential (-0.30 MPa) (**Figure 1**). The optical density declined as matric stress was increased, but all strains except DR11 maintained a similar cell density at -0.05 MPa to that observed under non-stressed conditions. It was surprising that *Pseudomonas fluorescens* DR7 reached a higher cell density at -0.05 MPa than was reached under non-stressed conditions, and maintained this maximum cell density at -0.3 MPa (**Figure 1**).

**FIGURE 2:**
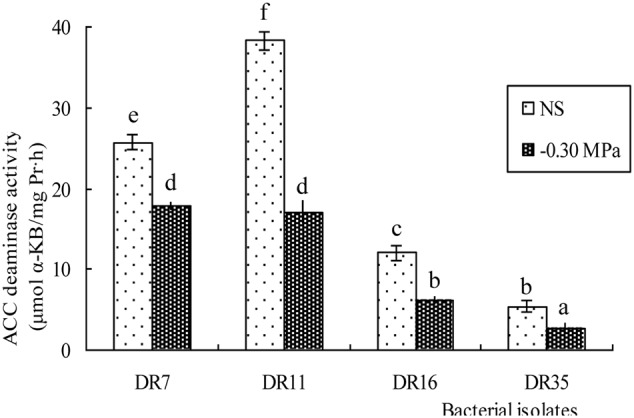
ACC deaminase activity in isolated bacteria under non-stressed (NS) and drought-stressed (–0.30 MPa) conditions. Values with different letters are significantly different according to Duncan’s multiple range test (*P* = 0.05). Error bars show standard deviations of mean values.

All four isolates were further assessed for ACC deaminase activity under both non-stressed and drought-stressed (-0.3 MPa) conditions. The ACC deaminase activity of all strains was lower under drought stress conditions, and was reduced by 30.42 - 55.38% (**Figure 2**).

### Amplification of the ACC Deaminase Gene

The ACC deaminase (*acdS*) gene was amplified using PCR using degenerate primers. The expected product of approximately 755 bp was observed with all four drought-tolerant isolates, confirming the results of ACC deaminase assays. A BLAST search was performed using the amplified sequence, and marked sequence similarity was observed with the *acdS* genes in GenBank (Supplementary Figure 1). Some conserved domains of ACC deaminase were also found in the translated amplified partial *acdS* sequences. The *acdS* partial sequences of *Pseudomonas fluorescens* DR7, *Pseudomonas fluorescens* DR11, *Enterobacter hormaechei* DR16, and *Pseudomonas migulae* DR35 were submitted to GenBank under accession no. KY352308, KY451713, KY352309, and KY451712, respectively.

**FIGURE 3:**
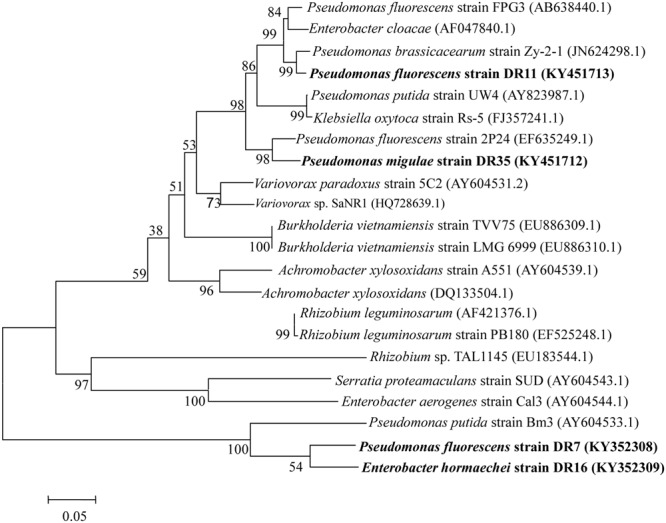
Phylogenetic analysis of the four ACC deaminase-producing bacteria based on *acdS* gene sequences available from the NCBI GenBank database. Distance and clustering analyses were performed using the neighbor-joining method using MEGA ver. 4.0. Bootstrap values (*n* = 1,000) are listed as percentages at the branching points.

Phylogenetic analysis of the partial sequences of the four isolates with existing sequences in the database revealed a significant polymorphism between these sequences. The generated phylogenetic tree revealed that ACC deaminase sequences of *Pseudomonas* isolate DR7 and *Enterobacter* isolate DR16 fell into the same clade as *Pseudomonas putida* strain Bm3 (AY604533.1), while DR11 and DR35 are more closely related to ACC deaminase sequences of other *Pseudomonas* species (**Figure 3**).

### Characterization of Other PGP Properties of the Isolates

The four drought-tolerant ACC deaminase-producing bacterial strains were tested for other PGP properties (**Table 2**). *Pseudomonas migulae* DR35 was found to produce significant amount of IAA (4.66 ± 0.05 mg/L). This isolate was also positive for amplification of the *nif*H gene, and grew on N-free semi-solid JNFb medium, suggesting that it has nitrogen fixation activity. *Pseudomonas fluorescens* DR7 and DR11 were positive for phosphate solubilization, as indicated by the appearance of well-developed clear zones on Pikovskaya’s agar medium with 2% tricalcium phosphate. All four strains were negative for siderophore production.

**Table 2:** Plant growth promoting traits of the isolated bacteria.

Isolates	Nitrogen	Phosphate	Siderophore	IAA production	EPS production
	fixation	solubilization	production	(mg/L)	(mg/mg protein)
DR7	–	+	–	–	11.63 ± 0.51c
DR11	–	+	–	–	2.91 ± 0.19a
DR16	–	–	–	–	5.44 ± 0.24b
DR35	+	–	–	4.66 ± 0.05	3.33 ± 0.29a

*Values are presented as the mean of replicates ± SD. Values followed by different lowercase letters are significantly different according to the Duncan’s range test (*P* = 0.05) in all treatments.*

All four strains showed mucoid growth on RCV-sucrose medium containing 40 g L^-1^ sucrose and the presence of capsular material under the microscope. These isolates were further assessed for EPS production. *Pseudomonas fluorescens* DR7 produced the largest amount of EPS (11.63 ± 0.51 mg/mg protein), followed by isolate DR16 (5.44 ± 0.24 mg/mg protein), DR35 (3.33 ± 0.29 mg/mg protein), and DR11 (2.91 ± 0.19 mg/mg protein) (**Table 2**).

### Effect of PGP Strains on Seed Germination under Drought Stress

Inoculation with *Pseudomonas fluorescens* DR7, *Pseudomonas fluorescens* DR11, *Enterobacter hormaechei* DR16, and *Pseudomonas migulae* DR35 increased the percentage of foxtail millet seeds germinating under drought stress conditions between -0.30 MPa and -1.03 MPa (**Table 3**). The seed germination percentage (both inoculated and control groups) was gradually reduced with increasing concentration of PEG 6,000. At a water potential of -0.49 MPa and -1.03 MPa, *Pseudomonas fluorescens* DR7 and *Pseudomonas fluorescens* DR11 showed significant seed germination-promoting activity (an enhancement of 13.68 - 141.82%). Meanwhile, *Enterobacter hormaechei* DR16 only showed a significant seed germination-promoting effect under severe drought stress (-0.49 MPa and -1.03 MPa), and *Pseudomonas migulae* DR35 didn’t affect the seed germination percentage significantly under the tested conditions. The roles of the bacteria in plant drought stress tolerance were further assessed using foxtail millet seedlings (discussed below).

**Table 3:** Effects of inoculation with the four ACC deaminase- and EPS-producing bacteria on foxtail millet seed germination.

Treatments	Non-stressed	–0.3 MPa	–0.49 MPa	–1.03 MPa
Not inoculated	96.00 ± 1.16a	88.67 ± 2.40ab	78.00 ± 2.31a	28.67 ± 4.06a
DR7	96.00 ± 1.16a	96.00 ± 2.16c	88.67 ± 1.76c	69.33 ± 3.53c
DR11	95.33 ± 1.33a	94.67 ± 1.33bc	89.33 ± 1.76c	47.33 ± 1.76b
DR16	98.00 ± 1.16a	88.00 ± 2.31ab	85.33 ± 0.67bc	41.33 ± 2.40b
DR35	97.33 ± 1.76a	84.00 ± 3.06a	80.00 ± 2.00ab	30.67 ± 4.06a

*Values are presented as the mean of replicates ± SD. Values followed by different lowercase letters are significantly different according to the Duncan’s range test (*P* = 0.05) in all treatments.*

### Growth of Foxtail Millet Seedlings Inoculated with PGP Strains

The four bacteria were used to inoculate 13-day-old foxtail millet seedlings, and the dry weight of seedlings treated with bacteria were compared with untreated control plants (**Table 4**). Under non-stressed condition, no significant growth difference was found between bacteria-inoculated and untreated seedlings apart from DR35. Drought stress severely affected the growth of foxtail millet seedlings, as reflected by the reduced dry weight of both inoculated and control seedlings under water stress conditions. However, inoculation of all strains significantly (*P* ≤ 0.05) increased the dry weight of stressed seedlings by 70.4 - 122.2% compared with non-inoculated plants, indicating the ability of inoculated bacteria to alleviate drought stress. Among the four strains, *Pseudomonas fluorescens* DR7 had the greatest effect, increasing the dry weight by 122.2%, consistent with its high ACC deaminase activity.

**Table 4:** Effects of inoculation with the four ACC deaminase- and EPS-producing bacteria on growth parameters of foxtail millet seedlings.

Treatments	Non-stressed			Drought-stressed		
	DW^a^	RAS/R^b^	Soil moisture (%)	DW	RAS/R	Soil moisture (%)
No inoculated	0.057a (±0.004)	34.31a (±3.17)	10.14a (±0.81)	0.027a (±0.005)	47.49a (±7.44)	3.03a (±0.20)
DR7	0.066ab (±0.013)	60.24c (±10.78)	19.80c (±3.32)	0.060c (±0.005)	79.53b (±10.06)	4.32b (±0.21)
DR11	0.060ab (±0.010)	41.21ab (±4.97)	11.63ab (±1.47)	0.044b (±0.006)	60.79ab (±6.17)	3.68ab (±0.91)
DR16	0.057a (±0.006)	48.89bc (±6.15)	13.41ab (±1.82)	0.047b (±0.007)	76.07b (±18.91)	3.78ab (±0.38)
DR35	0.076b (±0.012)	54.30c (±5.58)	14.63b (±1.05)	0.048b (±0.006)	65.97b (±7.36)	3.98ab (±0.52)

*^a^ Dry weight.*

*^b^ Root associated soil/root.*

*Mean values with different letters are significantly different at the 5% probability level (mean ± SD).*

Inoculation of *Pseudomonas fluorescens* DR7 and *Pseudomonas migulae* DR35 had a positive effect on SM, which was increased by 95.27 and 45.76%, respectively, under non-stressed conditions. However, only inoculation of *Pseudomonas fluorescens* DR7 significantly increased SM (by 42.57%) under drought-stressed conditions.

There was a positive effect of inoculation of *Pseudomonas fluorescens* DR7, *Enterobacter hormaechei* DR16, and *Pseudomonas migulae* DR 35 on the RAS/RT ratio, which was increased by 42.49 - 75.58% and 44.54 - 67.47%, respectively, under no stress and drought stress conditions, and the effect was positively correlated with EPS production (**Table 2**).

**FIGURE 4:**
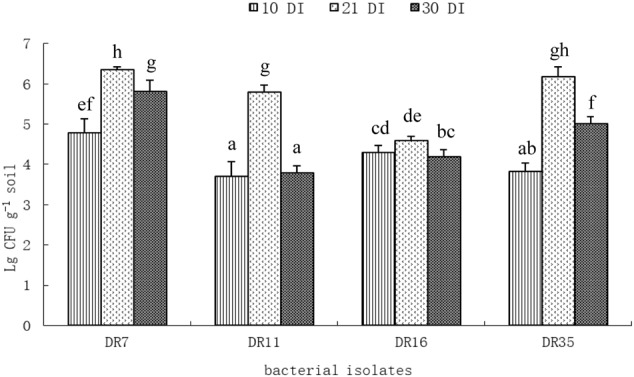
Population density of different bacteria inoculated to foxtail millet at different time intervals under axenic conditions. 10 DI, 10 days after inoculation; 21 DI, 21 days after inoculation; 30 DI, 30 days after inoculation (9 days after water stress). Values with different letters are significantly different according to Duncan’s multiple range test (*P* = 0.05). Error bars show standard deviations of mean values.

The population of inoculated bacteria in root-associated soil was assessed by colony counting on DF salts minimal agar medium supplemented with ACC as a sole nitrogen source. After 21 days of inoculation, all the bacterial inocula could colonized the rhizosphere successfully as indicated by significantly increased cell density (**Figure 4**). The population of *Pseudomonas fluorescens* DR7 and *Pseudomonas migulae* DR35 in RAS was up to 6.36 ± 0.06 and 6.18 ± 0.24 lg CFU g^-1^ soil. However, drought stress affected the colonization of inoculated bacteria in root-associated soil. In general, drought stress resulted in a significant decrease in rhizosphere bacteria population (**Figure 4**). After 30 days of inoculation (after 9 days of water stress), the population of *Pseudomonas fluorescens* DR7 and *Enterobacter hormaechei* DR16 in RAS decreased by 8.49 and 8.71%, respectively. However, the population sizes of *Pseudomonas fluorescens* DR11 and *Pseudomonas migulae* DR35 in rhizosphere decreased by 34.54 and 18.93%. Taken together, these results suggest that *Pseudomonas fluorescens* DR7 and *Enterobacter hormaechei* DR16 were more tolerant of drought stress than *Pseudomonas fluorescens* DR11 and *Pseudomonas migulae* DR35.

## Discussion

In this study, we isolated and characterized ACC deaminase-producing rhizosphere bacteria associated with millet foxtail. A total of 14 ACC deaminase-possessing strains producing 1.89–39.40 μmol α-KB/mg Pr⋅h were isolated from foxtail millet grown under semiarid conditions. Phylogenetic analysis of the 16S rRNA gene sequence revealed that they belonged to six genera: *Pseudomonas, Enterobacter, Pantoea, Klebsiella, Arthrobacter*, and *Ochrobactrum*. *Pseudomonas* was the most highly represented (six isolates). Although *Pseudomonas* sp. are widespread in agricultural soils and many have been widely studied for their enhancement of plant growth (Hol et al., 2013; Tiwari et al., 2016), the *Pseudomonas* isolates in our study exhibited higher ACC deaminase activity (3.24–39.4 μmol α-KB/mg Pr⋅h) compared with previously reported *Pseudomonas* from other crops (157–972 nmol α-KB/mg Pr⋅h) (Singh et al., 2015). Previous studies also found two *P. brassicacearum* strains with high level of ACC deaminase activity (8.65 and 9.39 μmol α-KB/mg Pr⋅h), which were associated with halophyte adapted to high-stress environment (Qin et al., 2014). The high ACC deaminase activity of these *Pseudomonas* strains might be related to the high-stress habitats of their host plants. Bacteria that have ACC deaminase activity help plants to withstand stress (biotic and abiotic) by reducing the level of stress ethylene.

Interestingly, isolates DR59 and DR 95 belong to the *Arthrobacter* genus of the *Actinobacteria*. The 16S rRNA gene sequence of isolate DR59 showed 100% similarity to that of *Arthrobacter siccitolerans* 4J27, a highly desiccation-tolerant, xeroprotectant-producing strain that was previously isolated from dry soil (Santacruzcalvo et al., 2013). Members of *Actinobacteria* exist in a complete spectrum of extreme ecosystems. The existence of acidtolerant, alkaliphilic, psychrotolerant, thermotolerant, halotolerant, alkalitolerant, haloalkalitolerant, and xerophilous *Actinobacteria* has been reported (Lubsanova et al., 2014). *Acidobacteria* was demonstrated to be a main component of the foxtail millet root microbiota by 16S rRNA gene amplicon sequencing (Jin et al., 2017). Although ACC deaminase activity is known to be present in various bacteria and some fungi, and *Actinobacteria* including *Streptomyces, Amycolatopsis, Mycobacterium*, and *Arthrobacter* have recently been reported to possess ACC deaminase activity and/or the *acdS* gene, and to enhance plant growth (Barnawal et al., 2014; Massimiliano et al., 2015; Singh et al., 2015), scarce study has been made to screen *Actinobacteria* from stress environment for their potential to produce ACC deaminase and to enhance plant growth. To the best of our knowledge, the present work is the first report of deaminase activity in an *Arthrobacter* species isolated from a drought-tolerant crop.

Four out of the 14 isolates were able to grow at a minimum water potential (-0.30 MPa). It was notably that all these drought tolerant strains were able to produce EPS. Tolerance of these bacterial strains to low osmotic levels (-0.30 MPa) and EPS-production in the present study were probably because of the naturalization in the semiarid habitats. EPS production has been suggested as a response to matric stress (Roberson and Firestone, 1992). Microbial EPS possesses unique water-retention and cementing properties, which not only protect bacteria against desiccation, but also protect host plants from drought stress through improved soil structure (Sandhya et al., 2009). Indeed, an increase in EPS production in *A. brasilense* Sp245 was deemed responsible for protection under extreme desiccation conditions (Konnova et al., 2001). The high tolerance of the four rhizobacteria to drought stress could be explained by production of EPS. EPS also helps bacteria to attach to and colonize plant roots via network of fibrillar material that permanently connects bacteria to the surface and prevents removal from the site (Bashan et al., 2004). Thus, inoculation of plants with EPS-producing rhizobacteria possessing multiple growth-promoting activities may improve efficacy of bacterial inoculants in arid or semiarid areas.

The ACC activity of *Pseudomonas fluorescens* DR11, *Enterobacter hormaechei* DR16, and *Pseudomonas migulae* DR35 under drought stress conditions were reduced by 55.38, 49.12, and 48.69% compared with non-stressed conditions, respectively. Special case was *Pseudomonas fluorescens* DR7, which exhibited the least ACC deaminase activity reduction (30.42%). This result was consistent with the high drought tolerance and EPS production of *Pseudomonas fluorescens* DR7. Nevertheless, all four strains maintained ACC deaminase activity between 2.75 and 17.86 μmol α-KB/mg Pr⋅h under drought stress conditions, it was reported that organisms with a level of ACC deaminase activity of 20 nmol α-KB/mg Pr⋅h or higher can promote host plant growth (Ali et al., 2014). The four drought-tolerant ACC deaminase-producing bacteria were further tested for their growth-promoting activity under drought stress. Treatment with these strains improved seed germination over the non-inoculated seeds of foxtail millet under different levels of drought stress, with DR7 and DR11 had the most prominent growth-promoting effects under high levels of drought stress (-0.49 MPa and -1.03 MPa), which was consistent with their ACC deaminase activity level. A similar improvement in seed germination under abiotic stress has been reported in other plants treated with ACC deaminase-producing PGPR (Bal et al., 2013; Qin et al., 2014). Inoculation of foxtail millet with any of the four drought-tolerant ACC deaminase-producing bacteria increased the seedling dry biomass significantly under drought stress conditions. Our results confirmed the findings of earlier work by other researchers who similarly demonstrated increased resistance to drought stress (Mayak et al., 2004; Zahir et al., 2008). Since we did not directly measured ethylene levels in our experiments, we do not know whether levels of ACC deaminase are sufficient notably compared to that of the plant ACO enzyme. However, it was reported that bacterial ACC deaminase can indeed alleviate the adverse effects of ethylene by breaking down the precursor of ethylene synthesis (Mayak et al., 2004). The growth-promoting activity of these strains might not be solely attributable to ACC deaminase, and further research is needed to elucidate the mechanisms involved. However, our results confirmed that bacterial strains associated with foxtail millet rhizosphere contribute to the drought adaptation of their host plants. Many plants growing naturally under chronically stressful conditions were reported to harbor beneficial microbial communities that protect against abiotic stress (Rodriguez et al., 2008; Jha et al., 2012; Qin et al., 2014). Rhizobacteria inhabiting sites where water is regularly limited by repeated dry periods are likely to be more adapted to matric stress and more able to promote plant growth than bacteria isolated from sites where water sources are more abundant (Mayak et al., 2004). Our present study agrees with previous findings that plants in high-stress environment are useful sources of stress tolerant bacteria with plant growth-promoting potential.

All strains except DR11 increased the RAS/RT ratio under drought stress conditions (**Table 4**). This is probably due to an aggregating effect of the EPS produced by the inoculated bacteria. Additionally, the population size of inoculated strain in the foxtail millet rhizosphere was positively correlated with EPS production in the inoculated bacteria. It was suggested that the bacterial EPS might provide a microenvironment that holds water and dries more slowly, thus protecting bacteria from drying (Sandhya et al., 2009). In the present study, isolate DR7 had the highest EPS production, showed best survival and persistence in the rhizosphere under drought stress conditions, which is consistent with its performance in liquid culture. Notably, inoculation of DR7 significantly increased SM under non-stressed and drought-stressed conditions, indicating that this strain has a positive effect on the retention of water in rhizosphere soil. Meanwhile, although isolate DR11 showed the highest ACC deaminase activity, it did not have an appreciable effect on the RAS/RT ratio or SM, and the rhizobacterial population size of inoculated DR11 decreased drastically under drought stress conditions (**Figure 4**). The plant growth-promoting efficacy of PGPR depends considerably upon their ability to survive and establish effective root colonization (Lugtenberg and Kamilova, 2009; Bulgarelli et al., 2013). Furthermore, effective colonization of plant roots by PGPR plays an important role in growth promotion irrespective of the mechanism of action (Davey and O’loole, 2000). Screening of PGPR should therefore be mindful of their ability to survive and colonize the rhizosphere of plants under adverse conditions such as dehydration.

In summary, our present study agrees with previous findings that plants in natural drought conditions are useful sources of drought-tolerant bacteria with plant growth-promoting potential. The results presented here also support the hypothesis that PGPR can contribute to the drought habitats adaptation of plants like millets. We suggest that ACC deaminase- and EPS-producing bacteria in our research could be useful for the development of bio-inoculants for abiotic stress management in plants.

## Conclusion

The present study suggests that foxtail millet plants cultured in arid land are naturally associated with a variety of rhizobacteria that exhibit high tolerance to drought stress and display plant growth-promoting traits. The roots of foxtail millet could therefore act as a resource for rhizobacteria that are capable of directly protecting plants from drought stress. Our results suggest that ACC deaminase- and EPS-producing bacteria associated with foxtail millet could alleviate drought stress in plants, as indicated by improved seed germination and seedling growth. Production of EPS by ACC deaminase-producing bacteria appears to improve their efficiency as PGP bacteria in drought environments, possibly by improving soil structure and colonization. These results also suggest that multiple PGP traits should be considered to identify more efficient PGPR inoculants for future use in agriculture.

## Author Contributions

XN conceived and designed the experiments. XN, WG, and LS performed the experiments. LS analyzed the data and co-wrote the paper. WG, YX, and XN contributed reagents/materials/analysis tools.

## Conflict of Interest Statement

The authors declare that the research was conducted in the absence of any commercial or financial relationships that could be construed as a potential conflict of interest.
