# Supplementary material for: Drought-Tolerant Plant Growth-Promoting Rhizobacteria Associated with Foxtail Millet in a Semi-arid Agroecosystem and Their Potential in Alleviating Drought Stress
Source: Front Microbiol. 2018 Jan 11;8:2580. doi: 10.3389/fmicb.2017.02580 (PMC5771373; doi:10.3389/fmicb.2017.02580)
Supplement: Supplementary file 1 [file Image_1.PDF]

```
PSEUDOMONAS_FLUORESCENS_STRAIN_DR7             KTRKLEYVIPEAIDQGCDTLVSIGGIQSNQTRQVAAVAAHLGMKCVLVQENWVNYSDAVYDRVGN    65
PSEUDOMONAS_FLUORESCENS_STRAIN_DR11            KMRKLEYLIPEALEQGCDTLVSIGGIQSNQTRQVAAVAAHLGMKCVLVQENWVNYSDAVYDRVGN    65
ENTEROBACTER_HORMAECHEI_STRAIN_DR16            KTRKLEYLIPEALEQGCDTLVSIGGIQSNQTRQVAAVAAHLGMKCVLVQENWVNYSDAVYDRVGN    65
PSEUDOMONAS_MIGULAE_STRAIN_DR35                KTRKLEYLIPEALEQGCDTLVSIGGIQSNQTRQVAAVAAHLGMKCVLVQENWVNYSDAVYDRVGN    65
VARIOVORAX_PARADOXUS_STRAIN_5C2                KTRKLEYLIPEALEGGYDTLVSIGGIQSNQTRQVAAVAAHLGLKCVLVQENWVNYSDAVYDRVGN    65
PSEUDOMONAS_PUTIDA_STRAIN_UW4                  KTRKLEYLIPEAIEQGCDTLVSIGGIQSNQTRQVAAVAAHLGMKCVLVQENWVNYSDAVYDRVGN    65
PSEUDOMONAS_BRASSICACEARUM_STRAIN_ZY-2-1       KTRKLEYLIPEALEQGCDTLVSIGGIQSNQTRQVAAVAAHLGMKCVLVQENWVNYSDAVYDRVGN    65
ACHROMOBACTER_XYLOSOXIDANS_STRAIN_A551         KLRKLEYLIPQALEQGCDTLVTIGGIQSNHTRMVAAVAAKLGLACVLVQENWVDYSDAVYDRVGN    65
BURKHOLDERIA_VIETNAMIENSIS_STRAIN_LMG_6999     KTRKLEYLIPDALAQGADTLVSIGGVQSNQTRQVAAVAAHLGMKCVLVQEHWVNYDDPVYDRVGN    65
RHIZOBIUM_LEGUMINOSARUM_STRAIN_PB180           KLRKLEYIVPDAIASGADTLVSIGGVQSNHTRMVAATAAKIGMKCVVIQEKWVPHYDAVYDRVGN    65
Consensus                                      k rkley  p a   g dtlv igg qsn tr vaa aa  g  cv  qe wv   d vydrvgn

PSEUDOMONAS_FLUORESCENS_STRAIN_DR7             IEMSRIMGADVRLDAAGFDIGIRPSWEKAMSDVRERGGKPFPIPAGCSEHPYGGLGFVGFAEEVR   130
PSEUDOMONAS_FLUORESCENS_STRAIN_DR11            IEMSRIMGADVRLDAAGFDIGIRPSWEKAMSDVVEQGGKPFPIPAGCSEHPYGGLGFVGFAEEVR   130
ENTEROBACTER_HORMAECHEI_STRAIN_DR16            IEMSRIMGADVRLDAAGFDIGIRPSWEKAMSDVVEQGGKPFPIPAGCSEHPYGGLGFVGFAEEVR   130
PSEUDOMONAS_MIGULAE_STRAIN_DR35                IEMSRIMGADVRLDAAGFDIGIRPSWEKAMNDVVERGGKPFPIPAGCSEHPYGGLGFVGFAEEVR   130
VARIOVORAX_PARADOXUS_STRAIN_5C2                IEMSRIMGADVRLDAAGFDIGIRQSWEQAMADVRAAGGKPFPIPAGCSEHPRGGLGSVGFAEEVR   130
PSEUDOMONAS_PUTIDA_STRAIN_UW4                  IEMSRIMGADVRLDAAGFDIGIRPSWEKAMSDVVERGGKPFPIPAGCSEHPYGGLGFVGFAEEVR   130
PSEUDOMONAS_BRASSICACEARUM_STRAIN_ZY-2-1       IEMSRIMGADVRLDAAGFDIGIRPSWEKAMSDVVEQGGKPFPIPAGCSEHPYGGLGFVGFAEEVR   130
ACHROMOBACTER_XYLOSOXIDANS_STRAIN_A551         IMMSRLMGADVRLVDQGFDIGFRRSWEEALEEVKRRGGKPYAIPAGASDHELGGLGYVGFAEEVR   130
BURKHOLDERIA_VIETNAMIENSIS_STRAIN_LMG_6999     IQLSRMMGADVRLVADGFDIGIRRSWEDAMESVRQAGGKPYPIPAGCSEHPLGGLGFVGFAEEVR   130
RHIZOBIUM_LEGUMINOSARUM_STRAIN_PB180           ILMTKLMGADSRLVEDGFDIGIRKSWEDAIQSVEDAGGKPYAIPAGASVHKFGGLGYVGFAEEVA   130
Consensus                                      i     mgad rl   gfdig r swe a   v   ggkp  ipag s h  gglg vgfaeev

PSEUDOMONAS_FLUORESCENS_STRAIN_DR7             QQEKELGFKFDYIVVCSVTGSTQAGMVVGFAADGRSKNVIGVDASAKPEQTKAQILRIARHTA     193
PSEUDOMONAS_FLUORESCENS_STRAIN_DR11            QQEKELGFKFDYIVVCSVTGSTQAGMVVGFAADGRSKHVIGIDASAKPEQTKAQILRIARHTA     193
ENTEROBACTER_HORMAECHEI_STRAIN_DR16            QQEKELGFKFDYIVVCSVTGSTQAGMVVGFAADGRSKHVIGIDASAKPEQTKAQILRIARHTA     193
PSEUDOMONAS_MIGULAE_STRAIN_DR35                EQEKQLGFKFDYIVVCSVTGSTQAGMVVGFAADGRSKNVIGIDASAKPEKTKAQILRIARHTA     193
VARIOVORAX_PARADOXUS_STRAIN_5C2                QQEAELGFKFDYLVVCSVTGSTQAGMVVGFAADGRADRVIGIDASAKPQQTFEQILRIAKNTA     193
PSEUDOMONAS_PUTIDA_STRAIN_UW4                  QQEKELGFKFDYIVVCSVTGSTQAGMVVGFAADGRSKNVIGVDASAKPEQTKAQILRIARHTA     193
PSEUDOMONAS_BRASSICACEARUM_STRAIN_ZY-2-1       QQEKELGFKFDYIVVCSVTGSTQAGMVVGFAADGRSKHVIGIDASAKPEQTKAQILRIARHTA     193
ACHROMOBACTER_XYLOSOXIDANS_STRAIN_A551         RPEADLGFKFDYIVVCAVTGSTQAGMVVGFAADGRADRVIGIDASATPEQTRAQVLRIARRTA     193
BURKHOLDERIA_VIETNAMIENSIS_STRAIN_LMG_6999     EQEAQLGFRFDYVVVCSVTGSTQAGMVVGFAADGRADRVIGIDASATPERTREQITRIARHTA     193
RHIZOBIUM_LEGUMINOSARUM_STRAIN_PB180           AQEKDLGFIFDYIIVCVVTGSTQGRMIVGFAALDRADRVIGIDASGTLQQTRNQVRKIVDATS     193
Consensus                                        e  lgf fdy  vc vtgstq  m vgfaa  r   vig das     t  q   i   t
```

Fig. 1. Multiple-sequence alignment of the deduced amino acid sequence of *acdS* gene from the four ACC deaminase-producing bacteria with the acdS proteins from *Variovorax paradoxus* strain 5C2 (AAT35829), *Pseudomonas putida* strain UW4 (AAV73804), *Pseudomonas brassicacearum* strain Zy-2-1 (AE062172), *Achromobacter xylosoxidans* strain A551 (AAT35835), *Burkholderia vietnamiensis* strain LMG 6999 (ACH81532) and *Rhizobium leguminosarum* strain PB180 (ABP88076). Identical amino acids are highlighted with a black background.
